# A Case Report of Babesiosis Seen Outside of its Endemic Area and Incubation Period

**DOI:** 10.7759/cureus.11926

**Published:** 2020-12-05

**Authors:** Jinal K Patel, Kiran Tirumalasetty, Bassem Zeidan, Parth Desai, Johnathan Frunzi

**Affiliations:** 1 Internal Medicine, Medical Center of Trinity, Trinity, USA; 2 Critical Care Medicine, Medical Center of Trinity, Trinity, USA

**Keywords:** babesiosis microti, ixodes scapularis tick, malaria like symptoms, endemic in northeast and upper midwestern, splenectomy, maltese cross, tick-borne infections, infectious and parasitic diseases, non-endemic region

## Abstract

*Babesia microti* is a parasitic alveolate that is usually transmitted by *Ixodes scapularis* tick, which also transmits Lyme disease. Babesiosis is endemic in the Northeast and Upper Midwestern regions of the United States. This case report illustrates a 29-year-old Hispanic male who presented to a Florida hospital emergency department with complaints of fever, generalized weakness, and flu-like symptoms over a duration of four days. Subsequently, he was diagnosed with babesiosis infection since he had a travel history to Cape Cod, Massachusetts about 10 weeks before presenting to the hospital. He was treated with atovaquone, clindamycin, and azithromycin. The importance of this report is to illustrate that babesiosis may occur outside its endemic area and incubation period.

## Introduction

*Babesia microti* is a parasitic alveolate that is usually transmitted by *Ixodes scapularis* ticks [1,2]. In the United States, the endemic areas of babesiosis include the Northeast and Upper Midwestern regions [1,2]. Due to its emerging health risk worldwide, clinicians must be aware of the several presenting manifestations of babesiosis such as fever, malaise, fatigue, vomiting, and jaundice [1,2]. Current therapy primarily consists of a combination of azithromycin and atovaquone, however, clindamycin and quinine may be administered in severe cases [1,2].

## Case presentation

A 29-year-old Hispanic male, with a history of hereditary spherocytosis treated with splenectomy at age three, who had traveled to Cape Cod, Massachusetts 10 weeks before presenting to a Florida hospital emergency department (ED) with fever, generalized weakness, and flu-like symptoms for the past four days. 

**Figure 1:**
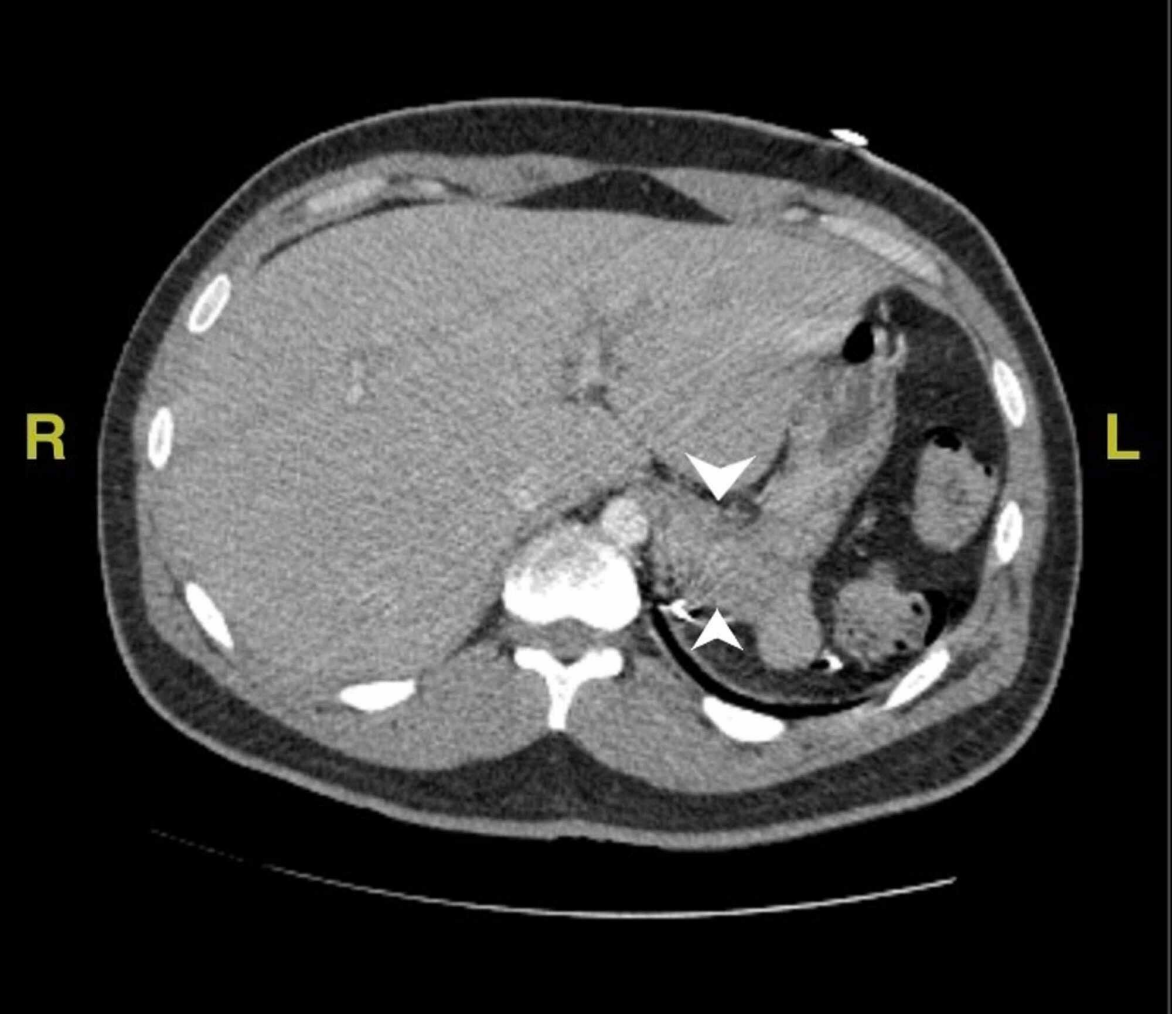
CT scan of the abdomen revealing peri-portal edema and hepatomegaly Peri-portal edema between the arrow heads Hepatomegaly as the liver crosses the midline

**Figure 2:**
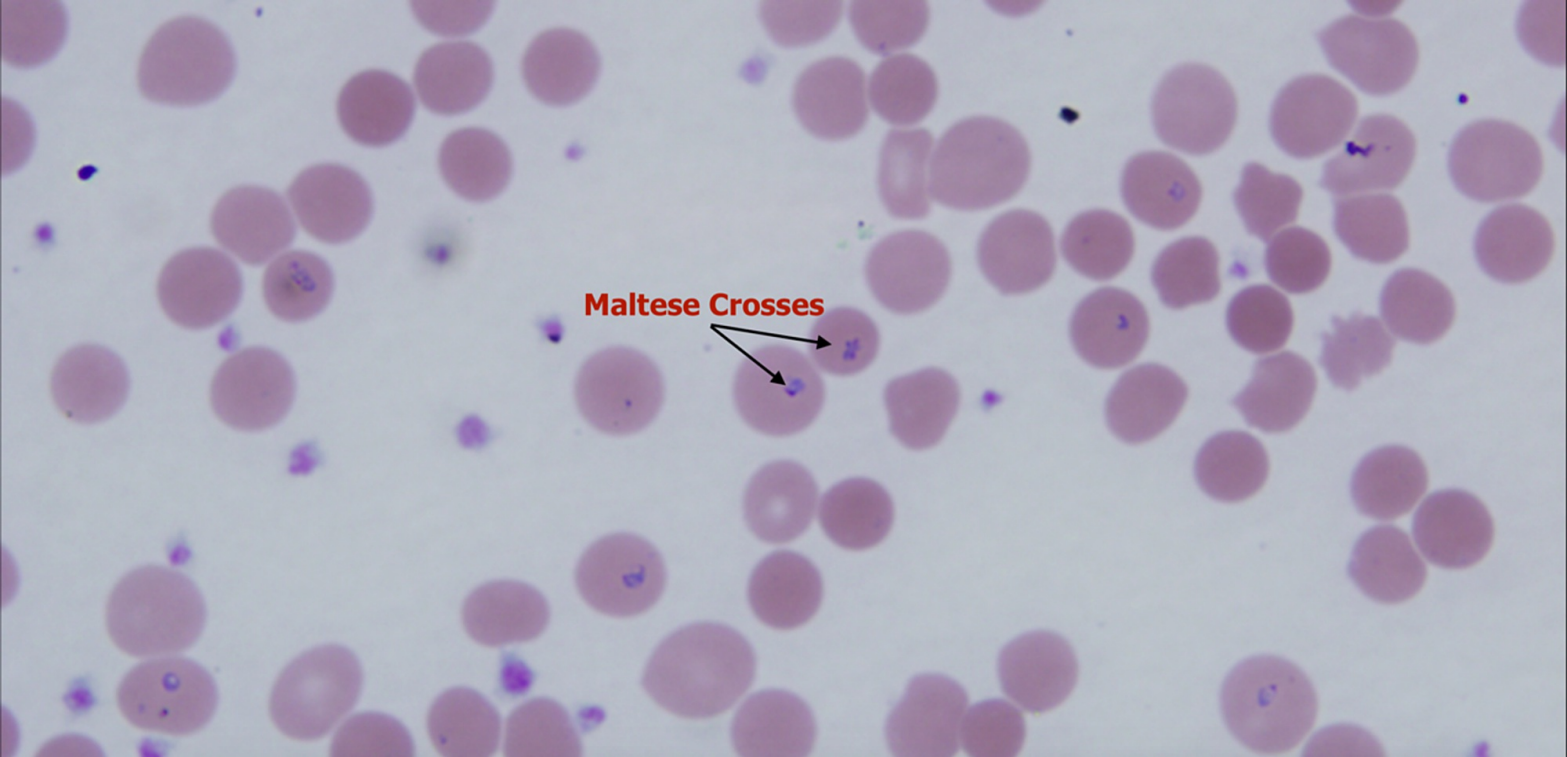
Peripheral smear showing the characteristic Maltese Crosses

On presentation, the patient was febrile to 104.8F. Initial laboratory investigation revealed hemoglobin (Hb) concentration of 9.3g/dL, which later dropped to 6.7g/dL within five hours after presenting to the ED. He was admitted to the intensive care unit (ICU) due to fever and suspected hemolysis. Patient was transfused with 2 units of packed red blood cells which increased his Hb to 7.8g/dL. Further laboratory workup for hemolysis revealed low haptoglobin (<20mg/dL), high serum lactate dehydrogenase (1035 unit/L), and high erythrocyte sedimentation rate (140 mm/Hr). A computerized tomography (CT) scan of the abdomen revealed some mild periportal edema suggestive of inflammation of the liver along with hepatomegaly (Figure 1). As there was no clear cause of his hemolysis, a peripheral blood smear (PBS) was obtained which was remarkable for maltese cross (Figure 2). This led to the final diagnosis of *Babesia microti* infection as he had traveled to an endemic region. 

The patient was initially treated with quinine but unfortunately experienced side effects including headache, tinnitus, and blurred vision. Subsequently, he was switched to atovaquone, clindamycin, and azithromycin, which he tolerated. The patient’s symptoms improved and he was discharged home with a seven-day course of oral clindamycin and azithromycin.

## Discussion

Infections with *Babesia microti* have been on the rise in the last couple of years [2]. National notifiable parameters added Babesiosis to the National Notifiable Conditions in 2011 which made the documentation of the disease further recognizable [2-4]. Although babesiosis is not considered a significant health concern in Florida, it was designated a reportable disease in 2017 in the state [2,3]. Our case was documented and the Centers for Disease Control and Prevention (CDC) was notified accordingly. 

Most cases of babesiosis may appear to be subclinical. However, symptomatic patients may present with nonspecific symptoms such as headache, muscle aches, fever, and fatigue which are more likely seen in asplenic or immunocompromised patients [4,5]. The average incubation period of babesiosis is typically one to nine weeks before symptoms manifest [1-3]. 

Babesiosis is most commonly misdiagnosed as a malaria-borne illness as both parasites, falciparum, and babesia are often seen in ring form within the red blood cells [4]. However, a Maltese cross seen on PBS is pathognomonic for babesiosis [4]. 

Our patient was initially thought to have been infected by the malaria parasite. However, he had traveled to Cape Cod, Massachusetts, an endemic area of *Ixodes* tick carrying *Babesia microti*, which allowed us to narrow down the differential between numerous tick-borne illnesses such as Lyme disease, Rocky Mountain spotted fever, and ehrlichiosis [3]. A case report by Stahl et al. [4] required polymerase-chain-reaction (PRC), whereas in our patient, we were able to see the pathognomonic maltese cross on PBS (Figure 2), to make the final diagnosis of babesiosis. Currently in high-risk patients (asplenic or immunocompromised) such as ours, current treatment consists of atovaquone and azithromycin or clindamycin. Quinine can be administered as an alternative treatment in severe cases along with blood transfusion [1,2].

## Conclusions

Clinicians should have a heightened awareness of babesiosis as it can present in nonendemic areas. Severe disease may occur in asplenic and immunocompromised hosts. This case report illustrates the importance of obtaining a thorough travel history during the initial encounter to recognize diseases outside of its endemic area and incubation period. 
